# Identification and validation of a prognostic-related mutant gene *DNAH5* for hepatocellular carcinoma

**DOI:** 10.3389/fimmu.2023.1236995

**Published:** 2023-10-25

**Authors:** Zebing Song, Xiaodong Song, Hang Li, Zongbing Cheng, Zengyi Mo, Xuewei Yang

**Affiliations:** Department of Hepatobiliary Surgery, The Second Affiliated Hospital of Guangzhou Medical University, Guangzhou, Guangdong, China

**Keywords:** hepatocellular carcinoma, TMB, mutant gene, DNAH5, immunotherapy target

## Abstract

**Background:**

Hepatocellular carcinoma (HCC) is one of the leading causes of cancer-related deaths worldwide and has a poor prognosis. Thus, there is a need for an effective biomarker to improve and predict the prognosis of HCC.

**Methods:**

RNA sequencing data, simple nucleotide variation data, and clinical data of HCC patients from The Cancer Genome Atlas (TCGA) to identify mutant genes, simple nucleotide variation data, and clinical data of HCC patients from the International Cancer Genome Consortium (ICGC) to validate the prognostic value of mutant genes were the data sources of the present study. To identify the overall survival (OS)-related mutant genes, a Kaplan–Meier (KM) analysis was conducted. We carried out univariate Cox and multivariate Cox regression analyses to identify the independent prognostic factors. We also conducted a correlation analysis of immune cells and mutant genes. To explore the molecular mechanisms of mutant genes, we conducted a gene set enrichment analysis (GSEA). A nomogram was constructed to help predict the prognosis of HCC. In addition, we explored the expression profile of mutant genes in HCC based on a TCGA dataset, an ICGC dataset, and our own HCC tissue samples.

**Results:**

We identified and validated a mutant gene, dynein axonemal heavy chain 5 (*DNAH5*), which was negatively related to the OS of HCC patients. Univariate Cox and multivariate Cox regression analyses revealed that the mutant gene *DNAH5* could act as an independent prognostic factor for HCC. Most pathways of the mutant gene *DNAH5* were involved in cancer development and progression based on GSEA analysis. The mutant gene *DNAH5* was negatively correlated with monocytes, naive CD4 T cells, activated dendritic cells, and activated mast cells. In addition, the mRNA and protein levels of *DNAH5* had a significantly higher level of expression in the tissue samples of patients with HCC. A nomogram consisting of the pathological stage, *DNAH5*, and tumor mutation burden (TMB) performed well.

**Conclusion:**

The mutant gene *DNAH5* had a significantly higher level of expression in the tissue samples of patients with HCC, could act as an independent prognostic factor for HCC, and is a potential new immunotherapy target for HCC.

## Introduction

Hepatocellular carcinoma (HCC), which has a background of chronic liver inflammation, is the sixth most common cancer and the cause of the third largest number of cancer-related deaths worldwide (1). The prognosis of HCC remains poor because of its high recurrence rate (2–4).

Recent advances in molecularly targeted therapy have helped improve the prognosis of patients with HCC, even those with advanced HCC. However, most HCC patients demonstrate tolerance to molecularly targeted drugs or become refractory to treatment during their clinical course (5, 6). Immunotherapy, on the other hand, is seen as a promising treatment approach, even for those patients with HCC who do not readily respond to traditional therapies (7).

It is widely believed that genetic mutations contribute greatly to the development and progression of cancer. Mutations in catenin beta 1 (*CTNNB1*) were negatively associated with the prognosis of HCC in the context of alcohol intake (8), while missense mutations of tumor protein p53 (*TP53*) were shown to be negatively associated with the prognosis of HCC in the context of hepatitis B virus infection (9). Previous studies have revealed that dynein axonemal heavy chain 5 (*DNAH5*) is a key gene in the development and progression of some cancers, including esophageal squamous cell carcinoma (10), melanoma (11), and colorectal cancer (12). However, the correlation between the genetic mutation of *DNAH5* and the prognosis of HCC is still unclear. In the present study, with the help of The Cancer Genome Atlas (TCGA) and the International Cancer Genome Consortium (ICGC) databases, we identified and validated an immunity-related mutant gene—*DNAH5*—that has a significant correlation with the biological function of immune cells and the prognosis of HCC in patients and may be a potential immunotherapy target for HCC.

## Materials and methods

### Data sources

**Figure 1:**
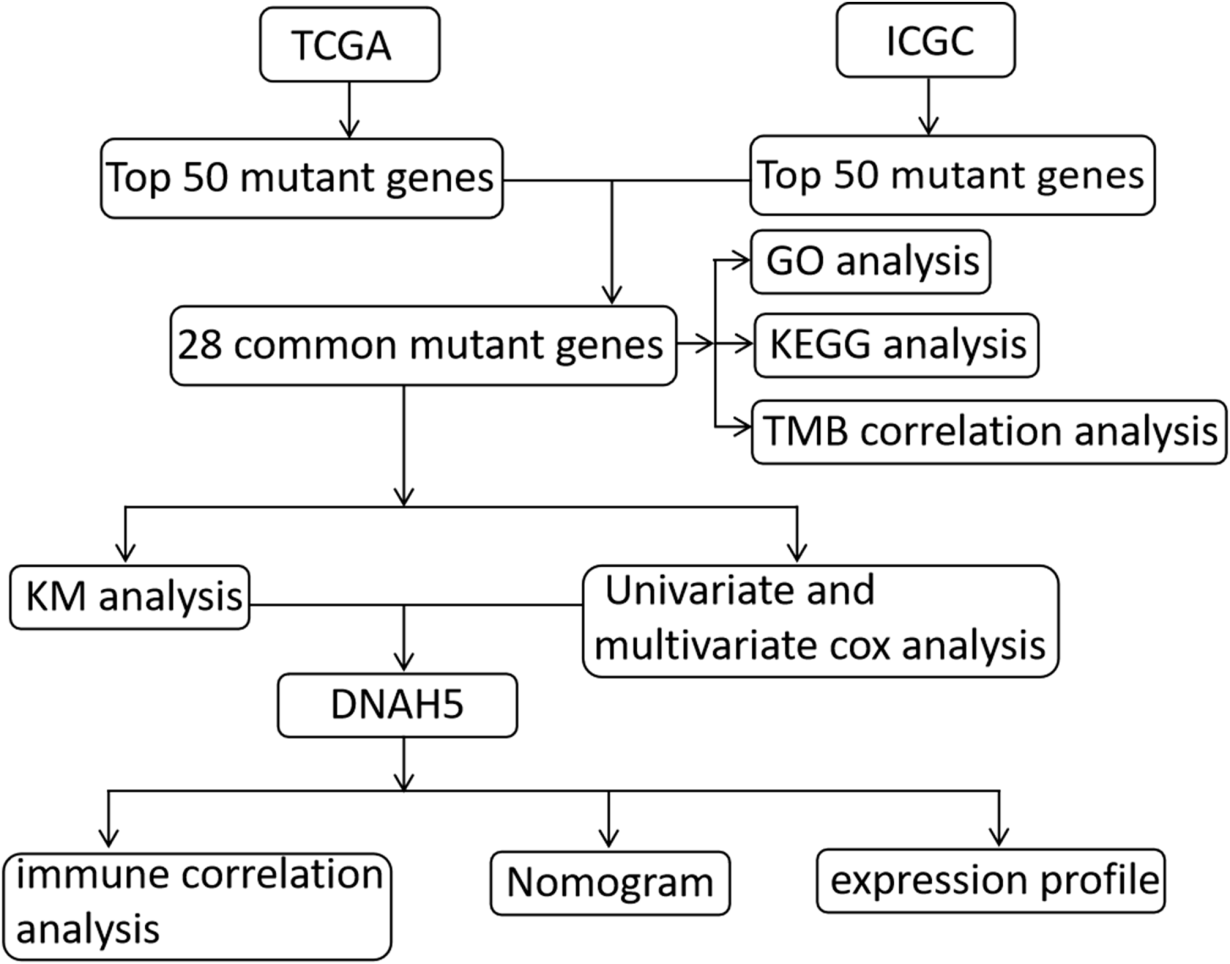
The flow chart of the present study.

The transcriptome profiling RNA sequencing data [i.e., high-throughput sequencing data—fragments per kilobase of exon per million fragments mapped (HTSeq-FPKM)], the simple nucleotide variation data calculated by SomaticSniper software, and the clinical data were taken from The Cancer Genome Atlas Liver Hepatocellular Carcinoma (TCGA-LIHC) data collection and were used to identify prognostic-related mutant genes. To validate the prognostic value of these mutant genes, simple nucleotide variation data and HCC clinical data were also downloaded from the International Cancer Genome Consortium Liver Cancer France (ICGC-LICA-FR) database. The patients in both databases with incomplete follow-up data were excluded. The ICGC and TCGA databases can be accessed without ethics approval as they are public databases; hence, we followed their guidelines for publication and data access policies in this study. A flow chart of the present study is shown in **Figure 1**.

### Gene mutation analysis and mapping the waterfall plot of mutant genes

With the help of Perl software (version 3.6.1), gene mutation analyses of the ICGC and TCGA datasets were conducted using simple nucleotide variation data. Only mutations that caused a change in the sequence of amino acids were regarded as gene mutations and included in the subsequent analysis. Subsequently, according to the mutation statistical analysis, the waterfall plot of mutant genes in the above two datasets was mapped to visualize the genetic mutation in HCC samples, respectively, using the GenVisR software package.

### Potential function of intersected mutant genes in the ICGC and TCGA datasets

To identify the prognostic-related mutant genes more accurately, the top 50 mutant genes of the ICGC and TCGA datasets were intersected. We conducted Gene Ontology (GO) and Kyoto Encyclopedia of Genes and Genomes (KEGG) pathway analyses by using the clusterProfiler R package to explore the potential biological function of these intersected intersected mutant genes, including their cellular components (CCs), biological processes (BPs), signaling pathways, and molecular functions (MFs).

### Tumor mutation burden analysis and correlation analysis between tumor mutation burden and intersected mutant genes

Tumor mutation burden (TMB) is defined as the number of mutations per 1 million bases. TMB analysis of the TCGA dataset was conducted using Perl software. In the present study, only a mutation that caused a change in the sequence of amino acids was regarded as a TMB and included in the subsequent analysis. The patients were categorized into the mutation group or the wild-type group based on the gene mutation analysis. The correlation analysis between TMB and intersected mutant genes (wild-type and mutation group) was conducted using the limma R package, and a box plot was mapped to visualize the difference using the ggpubr R package.

### Identification and validation of overall survival-related mutant genes

In the present study, the prognostic value of mutant genes was evaluated using the TCGA dataset and validated using the ICGC dataset. To identify the overall survival (OS)-related mutant genes, Kaplan–Meier (KM) analyses were conducted using the survival R package. Univariate and multivariate Cox analyses were conducted by utilizing the limma and survival R packages, and their clinicopathologic features and OS-related mutant genes were used to identify the HCC prognostic factors.

### Molecular mechanisms and immune correlation of OS-related mutant genes

We conducted gene set enrichment analysis (GSEA) to explore the potential molecular mechanisms of OS-related mutant genes. To calculate immunocyte population fractions, the CIBERSORT method (13) was used, which can distinguish 22 phenotypes of immune cells sensitively and accurately. The samples with a *p-*value of < 0.05 were considered significant. According to CIBERSORT results, the correlation analysis among OS-related mutant genes and 22 phenotypes of immune cells was carried out using the limma and vioplot packages.

### A nomogram for predicting the OS of patients with HCC

We developed a nomogram for predicting the 1-, 3-, and 5-year OS of patients with HCC by combining the prognostic-related factors selected from the univariate Cox regression analysis. We validated the predictive accuracy of the nomogram by comparing the observed actual probability with the calibration curve. The more consistent the reference line (black line) of the calibration curve (red line), the higher the accuracy of the nomogram.

### Tissue samples

A total of 24 HCC tissue samples and paired adjacent non-cancerous liver tissue samples were collected from patients who had undergone liver resection in The Second Affiliated Hospital of Guangzhou Medical University from May 2020 to December 2022. Before RNA and protein extraction, the samples were snap-frozen in liquid nitrogen and then stored at −80°C. This study was approved by the Ethics Committee of The Second Affiliated Hospital of Guangzhou Medical University (approval number 2023-ks-08).

### Quantitative real-time reverse transcription PCR

The TRIzol™ reagent was used to isolate total RNA from the tissue samples. The SuperScript™ First-Strand Synthesis System was used to reverse-transcribe complement DNA (cDNA). Subsequently, real-time PCR (RT-PCR) was conducted, in accordance with the manufacturer’s instructions, by using an RT-PCR system (LightCycler^®^ 480 Instrument II; Roche Holding, Basel, Switzerland). The primers for the glyceraldehyde 3-phosphate dehydrogenase (*GAPDH*) gene were used as an internal loading control. The primers used were: *DNAH5* (divergent primers), forward: 5′-GGACCTGGAGTTGCTGCTTGAC-3'; *DNAH5*, reverse: 5′-AGGCGTGCTGCTCATTTCTTCTAG-3'; *GAPDH*, forward: 5'-CTCCTAAACCATAGCACCATCG-3'; and *GAPDH*, reverse: 5′-CGTCAGTGCGGTCACAATC-3'. The 2^–ΔΔCT^ method was used to standardize all the values.

### Western blotting

Radioimmunoprecipitation assay (RIPA) lysis buffer was used to extract proteins from the tissue samples, and a bicinchoninic acid (BCA) kit was used to quantify the proteins. Sodium dodecyl-sulfate polyacrylamide gel electrophoresis (SDS-PAGE) was carried out using a 7.5% gel to separate the proteins. The separated proteins were transferred onto a polyvinylidene difluoride (PVDF) membrane. Subsequently, the PVDF membrane was blocked in 5% skim milk for 2 hours at room temperature and incubated at 4°C overnight with a polyclonal rabbit anti-*DNAH5* antibody (1:1,000; Invitrogen Life Technologies, Grand Island, NY, USA). The membranes were then incubated with horseradish peroxidase (HRP)-conjugated goat anti-rabbit secondary antibody (1:5,000; Abcam, Cambridge, UK) for 1.5 hours and visualized using the Immobilon™ Western Chemiluminescent HRP Substrate (Millipore, USA).

### Bioinformatic and statistical analysis

All bioinformatic and statistical analyses were conducted using R software (version 4.2.2) and Perl software (version 3.6.1). The genes with the top 50 mutation frequencies in the TGCA and IGGC databases were extracted using Perl. The R package “GenVisR” was used to visualize the mutations of these genes (14). These genes were intersected to obtain the genes with a high mutation frequency in both databases using the R package “venn.” The correlation analysis between the TMB and intersected mutant genes was assessed and visualized via the limma and ggpubr R packages, respectively. The GSEA analysis was conducted using the *DNAH5* mutation and expression matrix data in the GSEA software (v. 4.1.0) (15). The normalized enrichment score (NES) was calculated by setting the permutation values to 1,000, and a false-discovery rate (FDR) *p*-value < 0.05 was adopted to determine the evident enrichment pathways. CIBERSORT is a computational method that can assess the proportion of 22 immunocyte immune cells in tumor tissue based on transcriptome data (13). The matrix data of the immune cell proportion for each tumor sample were obtained using the CIBERSORT deconvolution algorithm, setting the filter condition to a *p*-value < 0.05. The matrix data visualization was conducted using the R package “corrplot”. The TCGA samples were assigned to either the wild-type group or the mutation group based on their *DNAH5* status. The difference analysis of immunocyte infiltration within the two groups was carried out using the R package “limma” and visualized using the R package “vioplot.” The survival curves were analyzed via KM survival analysis and evaluated using the log-rank test. The identification of prognosis risk factors was conducted through a survival analysis of the clinical characteristics of patients, including their age, gender, grade, stage, TMB, BCLC stage, and *DNAH5* classification via univariate and multivariate Cox regression analyses. The correlation between the mutant genes and TMB was evaluated using the Mann–Whitney *U*-test. For all comparisons, a two-tailed *p*-value < 0.05 was considered statistically significant.

## Results

### Gene mutation analysis of the TCGA and ICGC datasets

**Figure 2:**
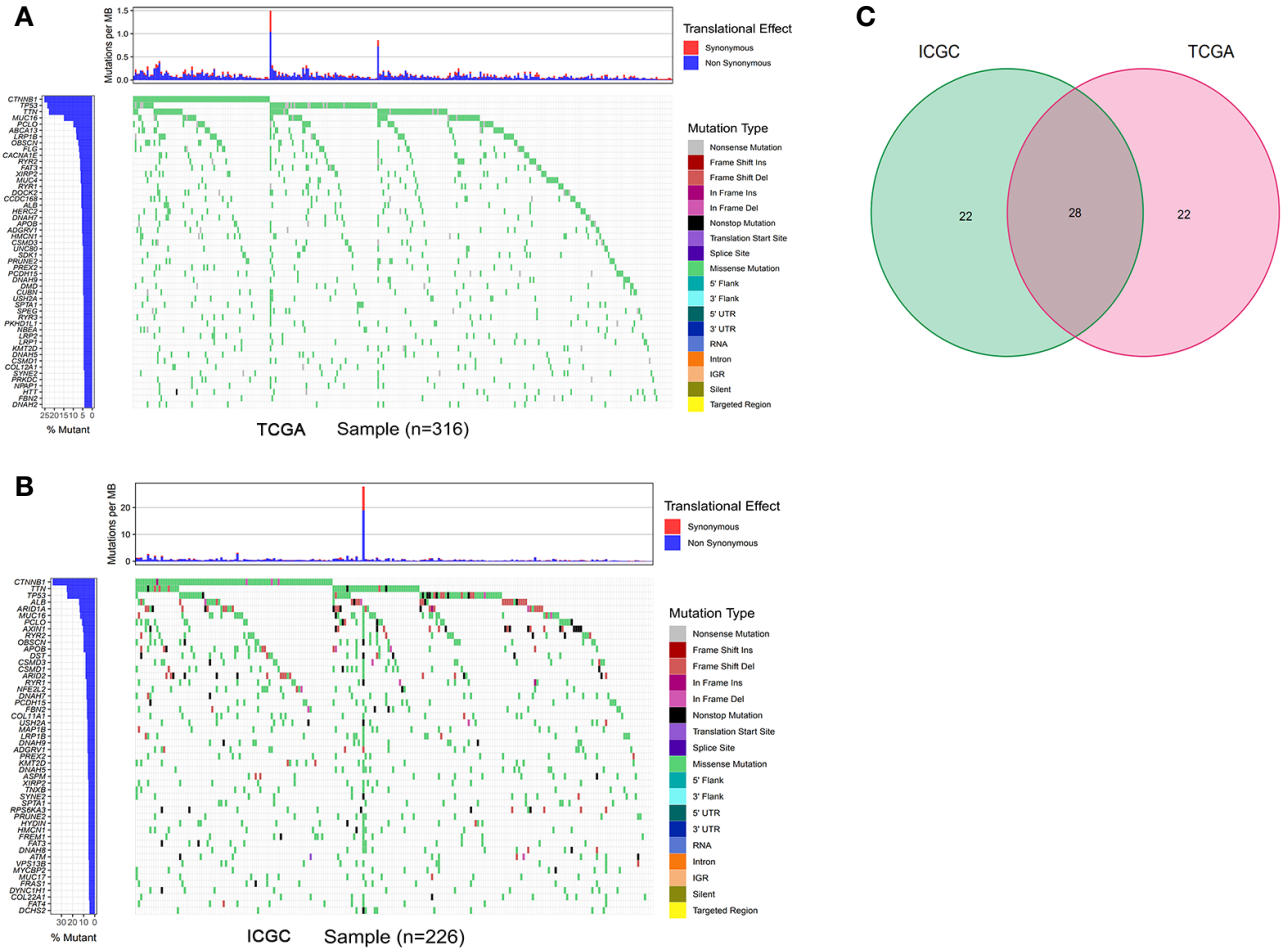
Gene mutation analysis of the TCGA and ICGC datasets. **(A, B)** The waterfall plot of the top 50 mutant genes of the TCGA and ICGC datasets, respectively. **(C)** intersected mutant genes of the TCGA and ICGC datasets. TCGA, The Cancer Genome Atlas; ICGC, International Cancer Genome Consortium.

**Figure 3:**
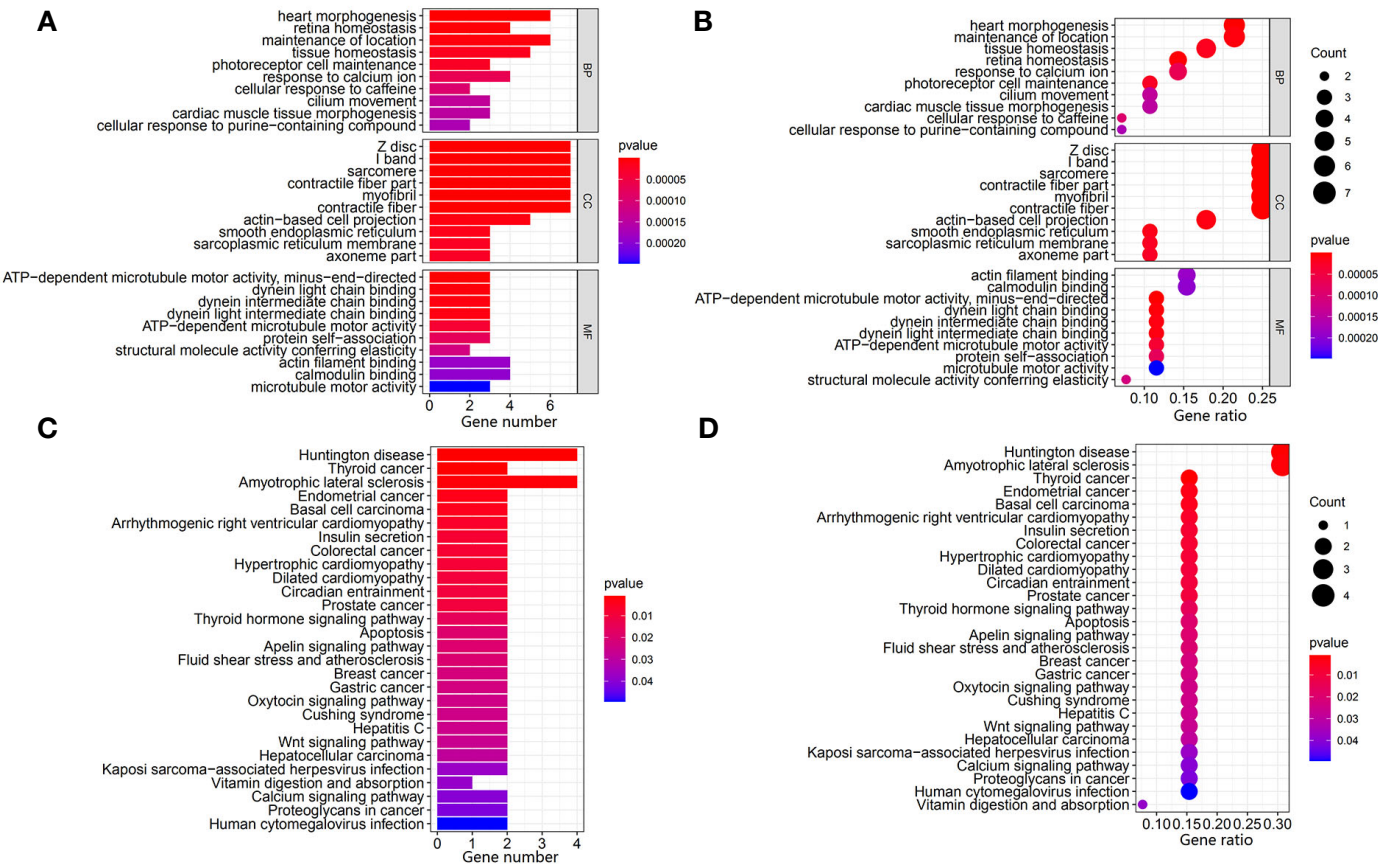
Identification and exploration of the potential function of intersected mutant genes in the TCGA and ICGC datasets. **(A, B)** Bar plot and bubble plot of the GO analysis of the intersected mutant genes, respectively. **(C, D)** Bar plot and bubble plot of the KEGG pathway analysis of the intersected mutant genes, respectively. TCGA, The Cancer Genome Atlas; ICGC, International Cancer Genome Consortium; GO, Gene Ontology; KEGG, Kyoto Encyclopedia of Genes and Genomes.

The gene mutation analysis showed that 10,981 and 11,098 mutant genes were identified in the ICGC and TCGA datasets. *CTNNB1*, *TNN*, and *TP53* were the top three mutant genes in both the ICGC and TCGA datasets. The waterfall plots of the top 50 mutant genes in the ICGC and TCGA datasets are shown in **Figures 2A, B**, respectively. A total of 28 mutant genes were identified in both the ICGC and TCGA datasets by way of intersected mutant gene analysis, which is shown in **Figure 2C**. The GO analysis showed that “maintenance of location,” “myofibril,” and “actin filament binding” were the most common functions of these mutant genes in terms of their BPs, CCs, and MFs, respectively (**Figure 3**). According to the KEGG analysis, these mutant genes were involved in the pathways of multiple cancers, including HCC, gastric cancer, breast cancer, and thyroid cancer (**Figure 3**).

### Identification and validation of OS-related mutant genes in HCC

**Figure 4:**
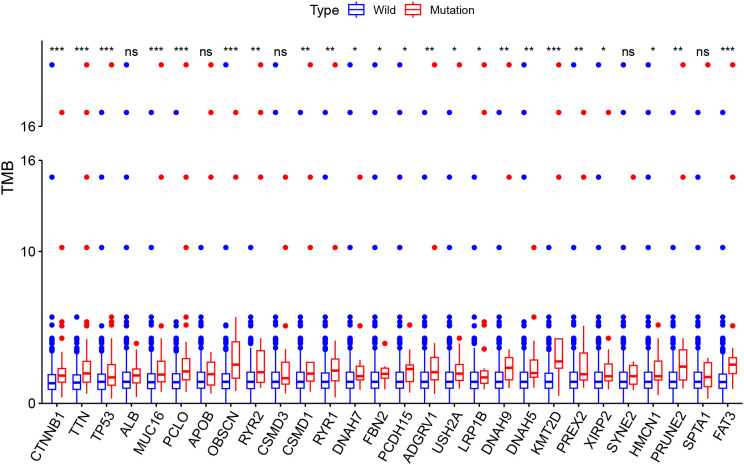
The correlation analysis between the TMB and intersected mutant genes. A total of 23 mutant genes had a higher TMB, and only five mutant genes had no correlation with the TMB. *p < 0.05; **p < 0.01; ***p < 0.001. TMB, tumor mutation burden; Ns, no signaficant.

**Figure 5:**
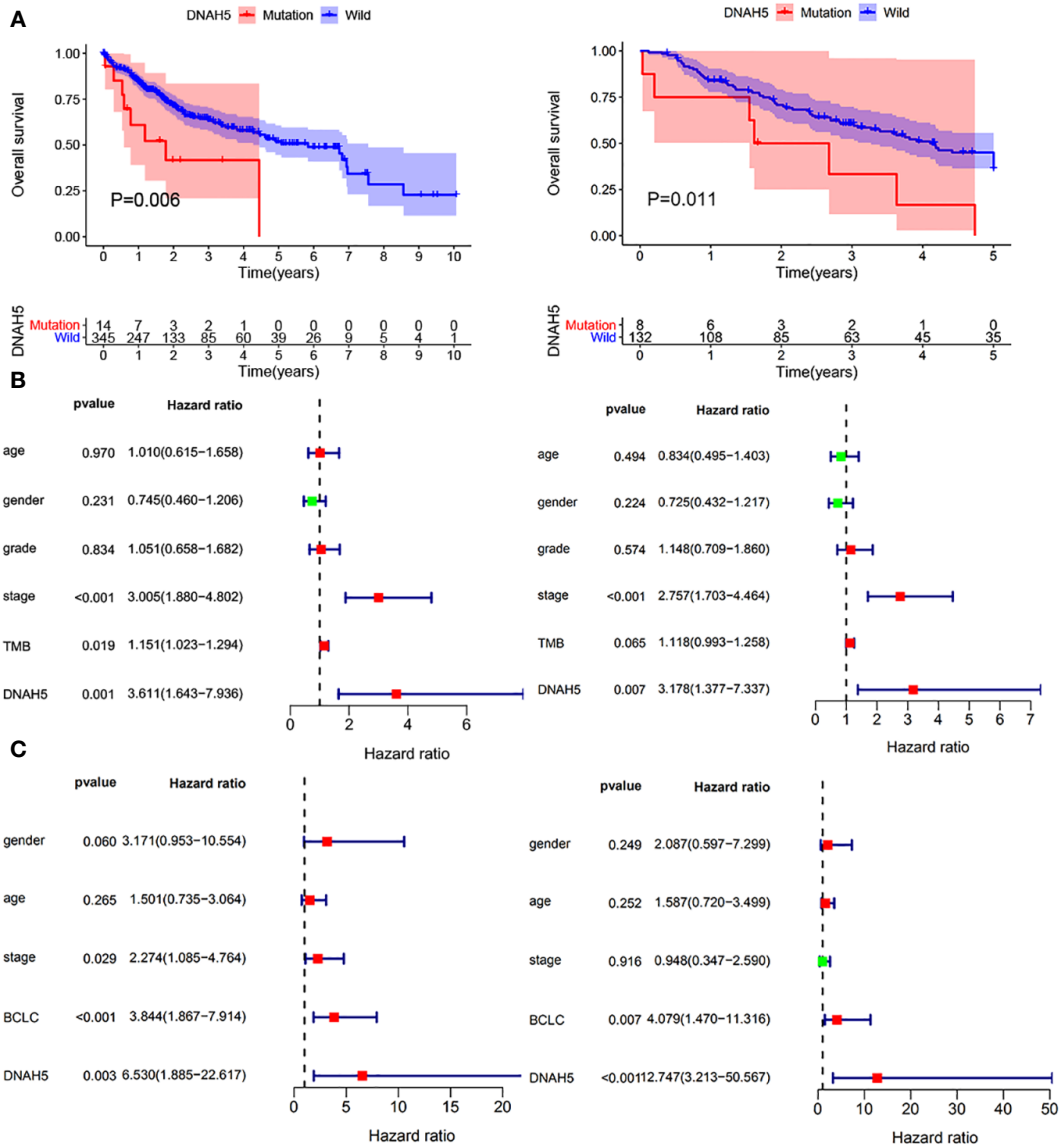
Evaluation of the prognostic value of the mutant gene *DNAH5* for HCC. **(A)** KM analysis of the OS of patients in the high-*DNHA5*-expression and low-*DNHA5*-expression groups in the TCGA dataset and ICGC dataset, respectively. **(B)** Univariate and multivariate Cox regression analyses of the mutant gene *DNAH5* and OS in the TCGA dataset. **(C)** Univariate and multivariate Cox regression analyses of the mutant gene *DNAH5* and OS in the ICGC dataset. TCGA, The Cancer Genome Atlas; ICGC, International Cancer Genome Consortium; OS, overall survival; HCC, hepatocellular carcinoma; KM, Kaplan–Meier.

The correlation analysis between TMB and intersected mutant genes showed that 23 mutant genes had a higher TMB, and only five mutant genes had no correlation with TMB, namely, albumin (*ALB*), apolipoprotein B (*APOB*), CUB and sushi domain-containing protein 3 (*CSMD3*), spectrin repeat-containing nuclear envelope protein 2 (*SYNE2*), and spectrin alpha, erythrocytic 1 (*SPTA1*); this is shown in **Figure 4**. To identify OS-related mutant genes, KM analysis of the above 23 TMB-related mutant genes was carried out. According to the results of the KM analysis, only patients with the *DNAH5* mutation had poorer OS than those without the *DNAH5* mutation in both the ICGC and TCGA datasets (**Figure 5A**). Univariate and multivariate Cox analyses of clinicopathologic features (including age, gender, tumor grade, pathological stage, and Barcelona Clinic Liver Cancer stage) and the mutant gene *DNAH5* were conducted to identify independent prognostic factors. Finally, *DNAH5* and pathological stage were independent prognostic factors in the TCGA dataset (**Figure 5B**), whereas in the ICGC dataset, *DNAH5* and Barcelona Clinic Liver Cancer (BCLC) stage were independent prognostic factors (**Figure 5C**).

### Exploration of the molecular mechanisms and immune correlation of the mutant gene *DNAH5*

**Figure 6:**
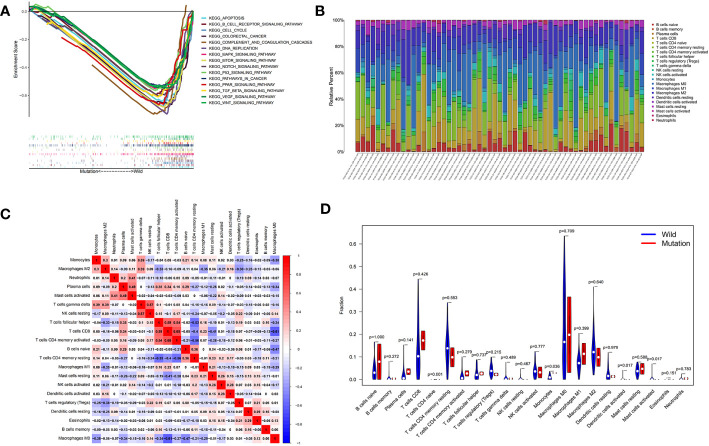
Exploration of the molecular mechanisms and immune correlation of *DNAH5* in HCC. **(A)** The main pathways in which mutant gene *DNAH5* was involved in based on the GSEA analysis. **(B)** Correlation matrix of immunocyte ratios in each TCGA data sample. **(C)** Correlation matrix of immunocyte ratios; the blue color represents the negative correlation and the red color represents the positive correlation. **(D)** Differentiated immunocyte infiltration between the mutant and the wild-type *DNAH5* groups; blue represents the wild-type *DNAH5* group, and red represents the mutant *DNAH5* group. HCC, hepatocellular carcinoma; TCGA, The Cancer Genome Atlas; GSEA, gene set enrichment analysis.

The GSEA (c2.cp.kegg.v7.symbol.gmt) revealed that the main biological functions of mutant gene *DNAH5* were related to cancer and that all 15 pathways were highly expressed in the wild-type group compared with the mutant group, namely, apoptosis, the B-cell receptor signaling pathway, complement and coagulation cascades, cell cycle, DNA replication, colorectal cancer, mitogen-activated protein kinase (MAPK) signaling pathway, vascular endothelial growth factor (VEGF) signaling pathway, mammalian target of rapamycin (mTOR) signaling pathway, wingless-related integration site (Wnt) signaling pathway, Notch signaling pathway, transforming growth factor beta (TGF-β) signaling pathway, p53 signaling pathway, pathways in cancer, and the peroxisome proliferator-activated receptor (PPAR) signaling pathway (**Figure 6A**). The highly expressed pathways of the mutant group contributed to promoting cancer development and progression, whereas the low-expressed pathways of the mutant group contributed to inhibiting cancer development and progression. The CIBERSORT method was conducted to explore the relationship between the mutant gene *DNAH5* and immune cells, as shown in **Figures 6B, C**. As a result, the mutant gene *DNAH5* was negatively related to naive CD4 T cells, monocytes, activated mast cells, and activated dendritic cells compared with those without a mutation of the *DNAH5* gene (**Figure 6D**).

### Exploration and validation of the gene expression profile of *DNAH5* in the tissue samples of patients with HCC

**Figure 7:**
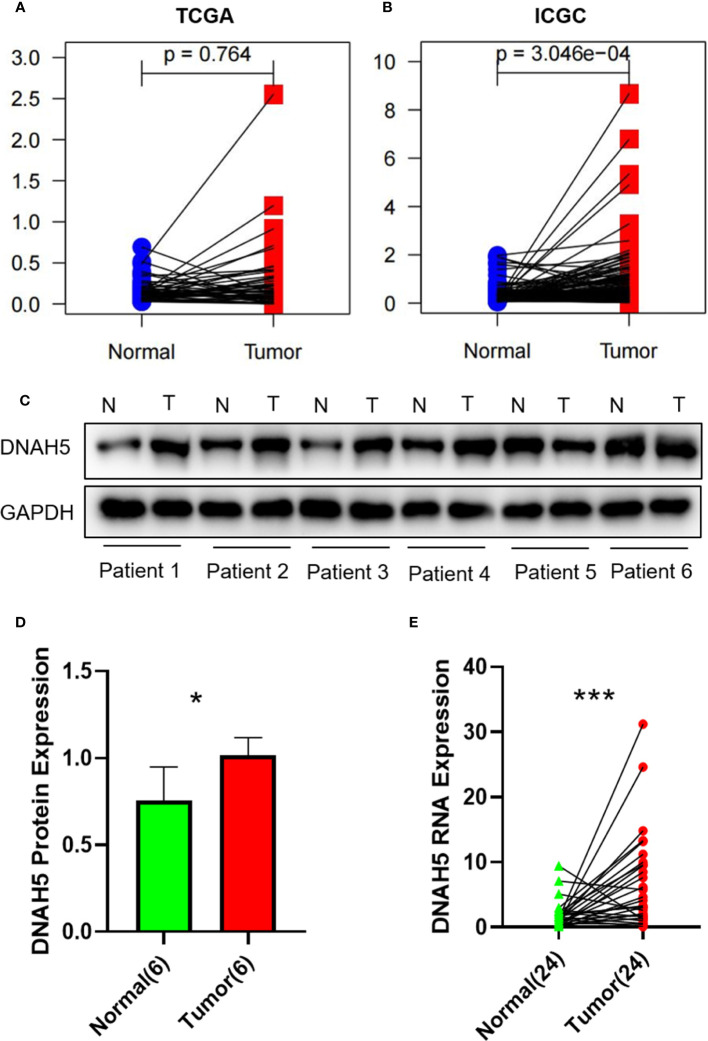
The gene expression profile of *DNAH5* in the TCGA dataset, ICGC dataset, and our own HCC tissue samples. **(A)** The mRNA expression profile of *DNAH5* in 50 pairs of HCC samples in the TCGA dataset. **(B)** The mRNA expression profile of *DNAH5* in 198 pairs of HCC samples in the ICGC dataset. **(C)** The protein expression profile of *DNAH5* in six pairs of HCC tissue samples and normal tissue samples based on Western blots. **(D)** A statistical graph showing the protein expression of *DNAH5* in six pairs of HCC tissue samples and normal tissue samples. **(E)** A statistical graph showing the mRNA expression of *DNAH5* in 24 pairs of HCC tissue samples and normal tissue samples. N, normal; T, tumor; TCGA, The Cancer Genome Atlas; ICGC, International Cancer Genome Consortium; HCC, hepatocellular carcinoma; mRNA, messenger RNA. *p<0.05; ***p<0.001.

We conducted paired differential expression analysis to explore the differential expression of *DNAH5* between HCC samples and non-tumor liver samples in the TCGA and ICGC datasets. A total of 50 paired tissue samples were included in the TCGA dataset and 198 paired tissue samples were included in the ICGC dataset. The results show that in the ICGC dataset, the level of mRNA expression of *DNAH5* in HCC samples was significantly higher than in the non-tumor samples, but no statistical difference was found in the samples from the TCGA dataset (**Figures 7A, B**).

Hence, to further explore the expression profile of *DNAH5* in HCC, the mRNA and protein expression profiles of *DNAH5* in HCC were validated in 24 pairs and 6 pairs of HCC tissue samples and adjacent non-cancerous liver tissue samples, respectively. As a result, the levels of mRNA and protein expression of *DNAH5* were significantly higher in the tissue samples of patients with HCC (**Figures 7C–E**). The clinical features of those patients are shown in **Supplementary Table 1**.

### Construction of a nomogram for predicting the OS of patients with HCC

**Figure 8:**
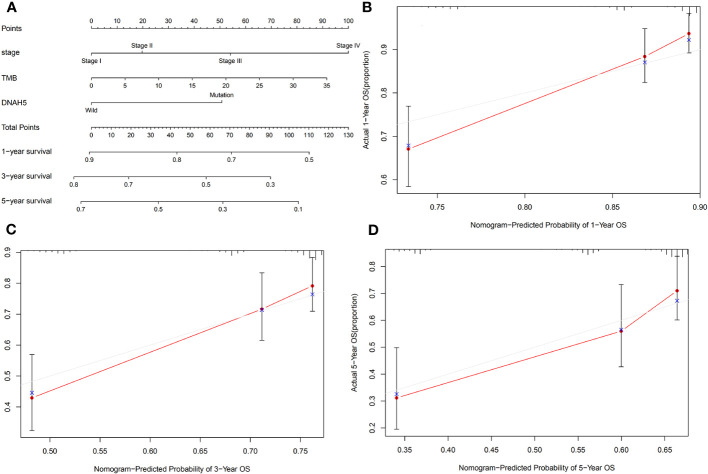
Construction of a nomogram for predicting the OS of patients with HCC. **(A)** A nomogram integrated the mutated gene *DNAH5* with pathological stage and TMB, which was able to predict the 1-, 3-, and 5-year OS of HCC patients. **(B–D)** The 1-, 3-, and 5-year OS calibration plots of the nomogram, respectively. The red dot represents the actual connection point of the calibration plots, and the blue cross represents an adjusted connection point of the calibration plots. OS, overall survival; HCC, hepatocellular carcinoma; TMB, tumor mutation burden.

We integrated the mutant gene *DNAH5* with other characteristics of HCC, including pathological stage and TMB, to develop a nomogram capable of predicting the 1-, 3-, and 5-year OS of HCC patients (**Figure 8A**). The calibration plots of this nomogram indicated that our nomogram performed well (**Figures 8B–D**).

## Discussion

As we all know, HCC is the fourth most common cancer and the cause of the third largest number of cancer-related deaths in China, and its incidence and mortality rates are 9.2% and 12.9%, respectively. Patients with HCC have a poor prognosis that is caused by its high post-treatment recurrence ratio.

Serum alpha-fetoprotein (AFP) levels have been used to diagnose HCC and predict its response to therapy over the past few decades. Nevertheless, other factors can affect the levels of AFP, including the cancer stage and tumor size, and as such, this technique lacks sufficient specificity and sensitivity to effectively diagnose HCC (16, 17). Recently, many new biomarkers, such as alpha-fetoprotein-L3 (AFP-L3), des-γ-carboxyprothrombin (DCP), Dickkopf-1 (DKKI), Golgi protein 73 (GP73), and Glypican-3 (GBC3), have attracted public attention. AFP-L3, as a heterogeneous body of AFP, mainly comes from HCC cells. DCP, also known as a protein induced by a lack of vitamin K or antagonist II (PIVKA-II), can appear in the serum of patients with a lack of vitamin K or with HCC (18). DKK1 is a secretory glycoprotein that inhibits the Wnt signaling pathway by binding to the Wnt receptor LRP5/6 (19). The Wnt signaling pathway is an important mechanism for the occurrence and development of HCC and other tumors. GP73, a type II transmembrane glycoprotein that is located in the Golgi apparatus, is expressed at a low level in normal liver tissue but can be highly expressed, especially around connective tissue and cirrhotic nodules, when liver diseases such as HCC occur (20). GPC3 is a heparan sulfate glycoprotein located on the surface of the cell membrane, which is a specific antigen related to HCC (21). However, these biomarkers lack sufficient specificity and sensitivity to effectively diagnose HCC. Therefore, it is important to identify some valid biomarkers to help guide the diagnosis and treatment of HCC.

In our study, we explored the role of the mutant gene *DNAH5* in HCC. Previous studies have revealed that *DNAH5* is a key gene in the development and progression of some cancers. According to the report by Qing et al., a genetic mutation in *DNAH5* contributes to a poorer prognosis of esophageal squamous cell carcinoma (10). In addition, Zhang and Xia reported that somatic mutations in *DNAH5* contribute to the genetic etiology of melanoma (11). Xiao et al. revealed that *DNAH5* was related to the development of colorectal cancer and might act as a biomarker for the diagnosis and treatment of colorectal cancer (12). However, the correlation between the genetic mutation of *DNAH5* and the prognosis of HCC is still unclear. In our present study, we revealed that a genetic mutation in *DNAH5* was negatively associated with the OS of HCC patients; this was validated in the ICGC dataset. In addition, univariate and multivariate Cox regression analyses of the ICGC and TCGA datasets showed that *DNAH5* was an independent prognostic factor for HCC. In addition, the expression profile of *DNAH5*, including mRNA and protein, was significantly higher in the HCC tumor tissue samples than in the normal tissue samples. Consequently, *DNAH5* might be a biomarker that could guide the diagnosis and therapy of HCC, and it is necessary that further studies validating the prognosis value of *DNAH5* in HCC are conducted.

TMB is defined as the number of mutations per megabase and has been recognized as a biomarker to predict the response to immune therapy (22). As we know, TMB contributes to a key point for developing and progressing of cancer (23). Endris V et al. showed that immune therapy responses were stronger in the high-TMB group than in the low-TMB group (24). Therefore, TMB is closely associated with the prognosis of cancer, and it is important to identify some of the key genes related to TMB (25). A univariate Cox regression analysis of the present study revealed that TMB was correlated with the OS of HCC patients, but that it showed no predicted values for the prognosis of HCC based on a multivariate Cox regression analysis, which may be because of the insufficient sample size. The present study revealed that a genetic mutation in *DNAH5* was significantly associated with the TMB of HCC as well. Due to *DNAH5*, the pathological stage and TMB were associated with the OS of HCC patients; consequently, we constructed a nomogram based on the pathological stage, *DNAH5*, and TMB to accurately predict the OS of patients with HCC. We validated the predictive accuracy of the nomogram using a calibration curve that showed moderate predictive accuracy compared with an ideal model. Therefore, the nomogram could act as a potential accurate predictive model for predicting the OS of patients with HCC.

Due to the limited information on the molecular mechanism of *DNAH5* in HCC, a GSEA analysis of KEGG pathways was performed. The results showed that most pathways were involved in cancer development and progression, such as the P53 signaling pathway, pathways in cancer, and the Notch signaling pathway. Our study provides potential directions for exploring the molecular mechanisms of *DNAH5* in HCC. Therefore, we hypothesize that the mutant gene *DNAH5* may act as a key gene in the prognosis of HCC by way of these pathways.

Targeted therapy and immunotherapy are two of the main treatment modalities for HCC patients, especially those with advanced HCC. Targeted drugs are mainly divided into small molecular tyrosine kinase inhibitors (TKIs), including sorafenib and lenvatinib, and macromolecular antibodies, such as antiangiogenic drugs, including bevacizumab and ranibizumab. Sorafenib and lenvatinib were approved in the Guidelines for the Diagnosis and Treatment of Primary Liver Cancer in China as maintenance therapies. Immune checkpoint inhibitors are mainly categorized as agents targeting programmed cell death 1 (PD-1), including nivolumab and tislelizumab; as agents targeting programmed cell death ligand 1 (PD-L1), including atezolizumab, durvalumab, and sintilimab; and as agents targeting cytotoxic T lymphocyte-associated antigen 4 (CTLA4), including ipilimumab and tremelimumab (26). However, targeted drugs and immunotherapy that treat HCC alone have limited efficacy in the treatment of HCC due to the tumor having a high degree of heterogeneity (27). Combined treatment with immune checkpoint inhibitors and TKIs has proven to be effective for HCC (28–30). Bevacizumab combined with atezolizumab and a bevacizumab analog combined with sintilimab have produced promising results as first-line therapies. Hence, to explore the potential correlation between the mutant gene *DNAH5* and immunotherapy for HCC, we explored the correlation between the mutant gene *DNAH5* and 22 phenotypes of immune cells. The results revealed that the mutant gene *DNAH5* was negatively correlated with naive CD4 T cells, monocytes, activated dendritic cells, and activated mast cells which indicated that the mutant gene *DNAH5* may affect the prognosis of HCC by inactivating the functions of immune cells. *DNAH5* may be a new potential immunotherapy target for HCC to improve the prognosis of patients with HCC. This study has some limitations; further *in vivo* and *in vitro* experiments are needed to validate its results, and further experiments are needed to explore the potential molecular mechanisms of *DNAH5* in HCC.

## Conclusion

The mutant gene *DNAH5* was more highly expressed in the tissue samples of patients with HCC and negatively associated with the OS of patients with HCC. As such, it may be a new potential immunotherapy target of HCC.

## Data availability statement

The original contributions presented in the study are included in the article/**Supplementary Material**. Further inquiries can be directed to the corresponding author.

## Ethics statement

This study involving humans was approved by the Ethics Committee of the Second Affiliated Hospital of Guangzhou Medical University (Approval number 2023-ks-08). This study was conducted in accordance with the local legislation and institutional requirements. The human samples used in this study were acquired primarily from subjects isolated as part of a previous study for which ethical approval was obtained. Written informed consent for participation was not required from the participants or the participants’ legal guardians/next of kin under the national legislation and institutional requirements.

## Author contributions

Conceptualization: ZS. Data curation: XY, XS, HL, ZC, and ZM. Data analysis and figure plots: ZS. Manuscript writing: ZS, XS, and XY. Article revision: XY. All authors contributed to the article and approved the submitted version.
