# Supplementary material for: Identification and validation of a prognostic-related mutant gene DNAH5 for hepatocellular carcinoma
Source: Front Immunol. 2023 Oct 25;14:1236995. doi: 10.3389/fimmu.2023.1236995 (PMC10630911; doi:10.3389/fimmu.2023.1236995)
Supplement: Supplementary file 1 [file DataSheet_1.docx]

The raw data of our manuscript has been transfered through jiang guo yun software and the shared data link is <https://www.jianguoyun.com/p/DUrFgFgQ37naCxi_wY0FIAA>
