# Supplementary material for: Identification and validation of a prognostic-related mutant gene DNAH5 for hepatocellular carcinoma
Source: Front Immunol. 2023 Oct 25;14:1236995. doi: 10.3389/fimmu.2023.1236995 (PMC10630911; doi:10.3389/fimmu.2023.1236995)
Supplement: Supplementary file 2 [file Table_1.docx]

Supplementary table 1. clinical features of 24 HCC patients

| **Parameters** | **Types** | **N=24** |
| --- | --- | --- |
| **age (year)** | <65 | 19 (79.2%) |
|  | >=65 | 5 (20.9%) |
| **gender** | male | 20 (83.3%) |
|  | female | 4 (16.7%) |
| **Tumor number** | single | 17 (70.8%) |
|  | multi | 7 (29.2%) |
| **Tumor diameter (cm)** | <=5 | 10 (41.7%) |
|  | >5 | 14 (58.3%) |
| **HBsAg** | yes  no | 15 (62.5%)  9 (37.5%) |
| **HCV** | yes | 2(8.3%) |
|  | no | 22(91.7%) |
| **AFP (μg/l)** | <=400 | 18 (75%) |
|  | >400 | 6 (25%) |
| **Albumin** | <35 | 3 (12.5%) |
|  | >=35 | 21 (87.5%) |
